# Quantitative evaluation of pregnant women delivery status’ records in Akure, Nigeria

**DOI:** 10.1016/j.dib.2017.11.041

**Published:** 2017-11-14

**Authors:** Adebowale O. Adejumo, Esivue A. Suleiman, Hilary I. Okagbue, Pelumi E. Oguntunde, Oluwole A. Odetunmibi

**Affiliations:** a Department of Mathematics, Covenant University, Ota, Nigeria; b Department of Statistics, University of Ilorin, Ilorin, Nigeria

**Keywords:** ARIMA, Caesarean section, Normal delivery, Data, Still birth, Time series, Akure

## Abstract

In this data article, monthly records (datasets) of total delivery, normal delivery, delivery through Caesarean section and number of still births from pregnant women in Akure, the capital city of Ondo state Nigeria, for a period of ten years, between January 2007 and December 2016 were considered. Correlational and time series analyses were conducted on the monthly records of total delivery, normal delivery (delivery through woman virginal), delivery through Caesarean section, and number of still births, in order to observe the patterns each of these indicators follows and to recommend appropriate model for forecasting their future values. The data were obtained in raw form from State Specialist Hospital (SSH), Akure, Ondo state, Nigeria. A clear description and variation in each of these indicators (total delivery, normal delivery, caesarean section, and still births) were considered separately using descriptive statistics and box plots. Different models were also proposed for each of these indicators using time series models.

**Specification Table**

**Table:** 

Subject area	Medicine
More specific subject area	Child Birth Delivery, epidemiology of delivery patterns, Biostatistics
Type of data	Table and figure
How data was acquired	Unprocessed secondary data
Data format	Processed as Monthly counts from 2007 to 2016 for Four different indicators on Child Birth Delivery
Experimental factors	Data obtained from State Specialist Hospital, Akure
Experimental features	Computational Analysis: Time Series Analysis, Time plot, ARIMA Models and Correlation Analysis.
Data source location	Ondo State Specialist Hospital, Akure, Ondo State, Nigeria
Data accessibility	All the data are in this data article
Software	R Statistical program and Microsoft Excel

**Value of the Data**

• The data on total delivery is a good indicator to monitor the population growth over the previous years.
• The data on still birth is a good indicator for the policy makers in the health sector to improve health facilities in the specialist hospitals and encourage pregnant women to attend anti-natal clinic regularly for necessary medical check-up.
• Data on still birth is also an indicator to create good access to maternal healthcare for all pregnant women at low or no cost.
• Data on still birth can be used to obtain still birth rate (SBR), post neonatal mortality rate (PNMR) and perinatal mortality rate (PMR) of a state or locality.
• Data on Caesarea Section is a good indicator for the government to encourage all pregnant women with any form of challenges on normal delivery to opt for Caesarea section with low or no cost in specialist hospitals.
• The data are for educational purposes and health assessment studies for example gynaecology, obstetrics, nursing and so on.
• The data on normal delivery can as well give a picture of whether there was improvement in the maternal healthcare in the previous years or not.
• The data is useful in the study of epidemiology of child delivery, computational gynaecology and public health studies.
• Several known models for example simple regression and probability fit can be applied to the data which provides alternative to analysis with time series. For example the use of linear, logistic or Poisson regression.

## 1 Data

The data for this paper was obtained from Ondo State Specialist Hospital, Akure, Ondo State, Nigeria. The data are on monthly total delivery, normal delivery, still birth, and delivery by Caesarean Section of pregnant women in the government owned State Specialist Hospital Akure, the capital city of Ondo State, for ten years; between January 2007 and December 2016.

Statistical summary of the monthly averages for each of the indicators (total delivery, normal delivery, still birth and Caesarean section) from January 2007 to December 2016 was given in Table 1. It was observed that the highest monthly total delivery of 436 were recorded in March 2010, while the highest monthly counts for still birth of 29, were recorded in both January and July 2008. However, in terms of proportion, the highest of 0.08815 (8.82%) were recorded in July 2008. Yearly total still births was 158 in 2007 and reduced to 30 in 2016, which amounts to 81% reduction in ten years. In addition, the highest number of Caesarean section of 64 was recorded in both October 2007 and February 2010.

**Table 1:** Summary statistics for the four delivery indicators for pregnant women in Akure.

Indicators	Minimum	1st Quartile	Median	Mean	3rd Quartile	Maximum
Total delivery	107.0	236.80	270.00	275.90	303.20	436.00
Normal delivery	90.00	208.00	241.50	242.00	269.80	383.00
Still birth	1.00	2.00	4.50	7.99	12.00	29.00
Caesarean section	7.00	25.00	33.00	33.87	41.00	64.00

Correlational results were shown in Table 2 and the result of the time series analysis is contained in Table 3, Table 4, Table 5, Table 6.

**Table 2:** 4×4 correlation matrix for the four indicators.

Indicators	Total delivery	Normal delivery	Still birth	Caesarean section
Total delivery	1			
Normal delivery	0.98098	1		
Still birth	0.62108	0.64250	1	
Caesarean section	0.60594	0.43990	0.24032	1

**Table 3:** ARIMA output for total delivery of pregnant women in Akure.

Model	ARIMA(0,1,1)		
Parameter	MA1		
Coefficients	−0.6238		
Standard error	0.0719	RMSE	42.6400
σ^2^ estimate	1834	Log-likelihood	−616.1800
AIC	1236.3700	BIC	1241.9300

**Table 4:** ARIMA output for normal delivery of pregnant women in Akure.

Model	ARIMA(0,1,1)		
Parameter	MA1		
Coefficients	−0.6222		
Standard error	0.0713	RMSE	37.0900
σ^2^ estimate	1399	Log-likelihood	−599.6000
AIC	1203.2000	BIC	1208.7600

**Table 5:** ARIMA output for still birth delivery by pregnant women in Akure.

Model	ARIMA(0,1,1)		
Parameter	MA1		
Coefficients	−0.6806		
Standard error	0.0667	RMSE	4.2200
σ^2^ estimate	17.9900	Log-likelihood	−341.1000
AIC	686.2100	BIC	691.7700

**Table 6:** ARIMA output for delivery of pregnant women through Caesarean section in Akure.

Model	ARIMA(3,0,0)					
Parameter	AR1	AR2	AR3	Mean		
Coefficients	0.1208	0.1183	0.2057	33.9664		
Standard error	0.0892	0.0935	0.0935	1.9175	RMSE	11.8300
σ^2^ estimate	144.7000				Log-likelihood	−466.8100
AIC	943.6200				BIC	957.5500

The raw monthly data for the aforementioned indicators are presented in Table 7, Table 8, Table 9, Table 10.

**Table 7:** Total monthly delivery of pregnant women between 2007 and 2016.

Month/Year	2007	2008	2009	2010	2011	2012	2013	2014	2015	2016
January	350	342	257	425	259	165	270	232	281	255
February	340	335	240	357	191	245	229	216	202	203
March	306	395	303	436	243	223	299	212	266	238
April	340	379	335	372	229	249	292	290	254	270
May	270	353	305	362	107	278	317	236	291	268
June	287	341	390	286	206	260	255	258	268	270
July	357	329	367	296	206	236	237	276	282	276
August	265	281	302	243	170	210	260	262	262	266
September	370	289	316	256	186	286	268	247	290	275
October	353	357	402	277	213	334	298	286	294	298
November	304	283	357	227	215	259	257	196	225	206
December	301	236	252	196	219	223	182	277	232	251

**Table 8:** Total monthly normal delivery of pregnant women between 2007 and 2016.

Month/Year	2007	2008	2009	2010	2011	2012	2013	2014	2015	2016
January	311	316	208	366	240	150	242	202	257	229
February	277	293	229	293	168	213	208	184	181	182
March	296	355	275	383	208	203	263	174	246	210
April	307	332	293	324	200	224	261	255	219	237
May	245	307	252	305	90	239	277	206	256	231
June	278	312	338	256	168	228	211	229	258	243
July	299	306	316	246	167	215	200	233	257	245
August	256	247	252	205	146	188	220	230	238	234
September	317	266	277	216	160	252	226	219	259	239
October	289	314	352	249	189	293	241	246	255	250
November	259	268	309	189	186	222	223	155	192	173
December	252	220	245	174	203	195	159	248	198	223

**Table 9:** Monthly number of pregnant women still birth between 2007 and 2016.

Month/Year	2007	2008	2009	2010	2011	2012	2013	2014	2015	2016
January	11	29	12	20	7	1	1	9	5	3
February	8	23	8	14	2	3	4	4	3	3
March	18	22	15	17	10	2	4	6	2	3
April	8	22	25	5	7	1	6	1	3	4
May	20	21	18	6	5	8	6	2	3	4
June	12	28	16	12	1	6	1	3	1	1
July	19	29	17	3	3	4	2	1	4	3
August	8	11	26	9	2	4	4	3	1	2
September	8	17	17	2	4	10	1	1	3	2
October	13	26	14	8	8	8	4	1	2	3
November	18	21	15	6	2	1	2	1	1	1
December	15	11	12	2	4	9	1	2	2	1

**Table 10:** Monthly number of pregnant women with Caesarean section between 2007 and 2016.

Month/Year	2007	2008	2009	2010	2011	2012	2013	2014	2015	2016
January	39	26	49	59	19	15	28	30	24	26
February	63	42	11	64	23	32	21	32	21	21
March	10	40	28	53	35	20	36	38	20	28
April	33	47	42	48	29	25	31	35	35	33
May	25	46	53	57	17	39	40	30	35	37
June	9	29	52	30	38	32	44	29	10	27
July	58	23	51	50	39	21	37	43	25	31
August	9	34	50	38	24	22	40	32	24	32
September	53	23	39	40	26	34	42	28	31	36
October	64	43	50	28	24	41	57	40	39	48
November	45	15	48	38	29	37	34	41	33	33
December	49	16	7	22	16	28	23	29	34	28

**Fig. 1:**
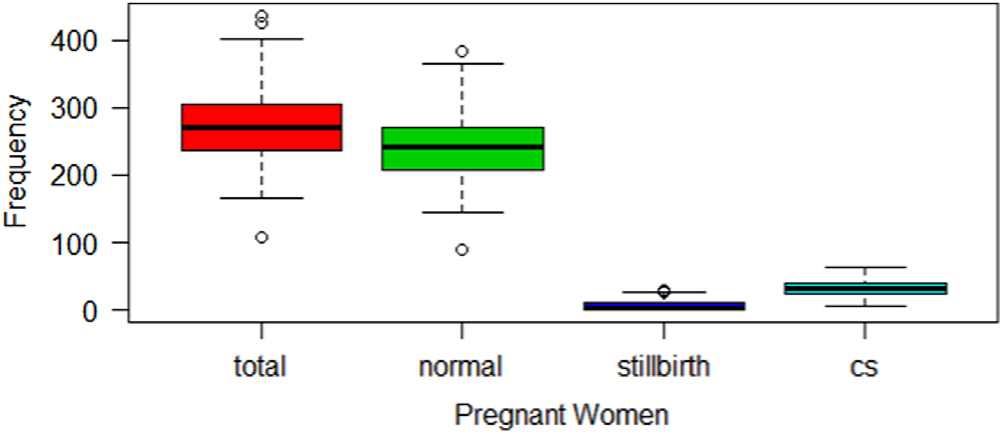
Boxplot for the four indicators on delivery of pregnant women in Akure.

The boxplot in Fig. 1 gives the description and variation in each of the indicators examined in this work. It shows that total and normal deliveries are very close to one another, as well as still birth and caesarean section. The boxplot is a chart presentation of Table 1, with extreme cases of delivery, evident from the outliers above and below each box representing the indicators, except for caesarean section (CS), which possesses no outlier.

**Fig. 2:**
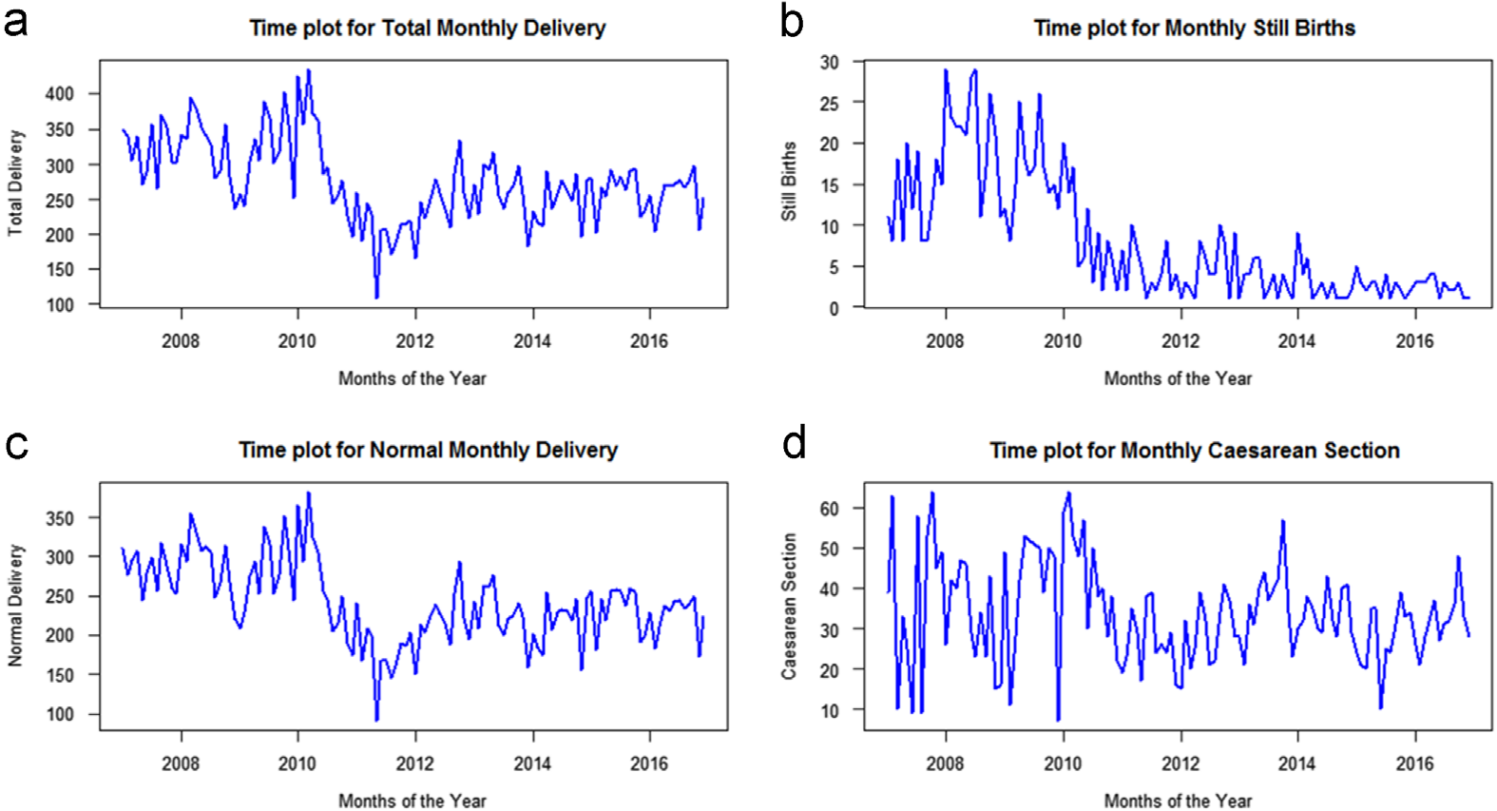
Time plots showing delivery states of pregnant women in Akure between 2007 and 2016.

Time Plot for each of the indicators in this paper is presented in Fig. 2a, b, c and d. This is designed to reveal the patterns observed in the given time interval.It can be observed from Fig. 2a and c that the total monthly and normal deliveries of pregnant women across the years under consideration were almost the same pattern.

The progression of pregnant women having still births, dropped drastically when compared with past years (2007–2009) as shown in Fig. 2a, b, c. The focus is on the trend and not on the year's interval.

Between 2014 and 2016, a steady trend was observed, which was stationary. This obviously resulted to the series being constant over studied time frame (period). In Fig. 2d, a trend surfaces between 2010 and 2016 which declines in the first month of every year. Furthermore, the number of pregnant women who underwent Caesarean section, from 2008 to 2016 is evidently declining, which could likely indicate the increasing fear of pregnant women and most especially the cost of being subjected to such mode of delivery.

**Fig. 3:**
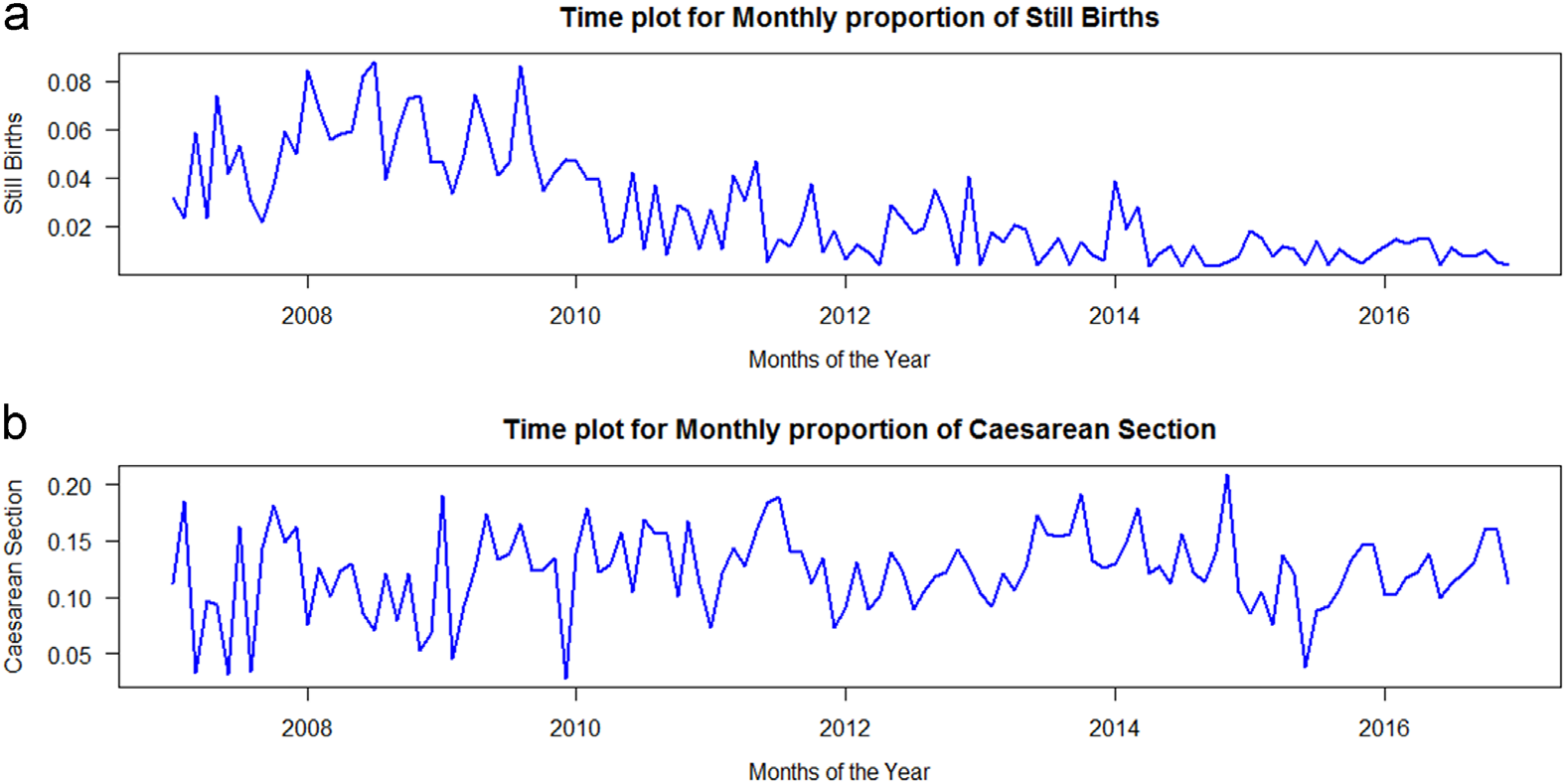
Monthly proportion for still birth and Caesarean delivery by pregnant women in Akure between 2007 and 2016.

It was observed from Fig. 3a, that the proportion of still birth dropped drastically towards year 2016, when compared with the first two or three years under consideration, that is from 2007 to 2009. It was also observed that, the total number of still births in year 2016 (30) was almost the same as the highest monthly (29) earlier recorded in both January and July 2008 respectively. This may be attributed to government efforts in the state to improve maternal and child healthcare is yielding dividends which eventually reduced the rate of monthly still birth in the state to the point of one or even zero as times goes on. The differences in the proportion of pregnant women undergoing Caesarean section across the years under investigation are not significant in pattern as seen in Fig. 3b. Furthermore, the plot showed that within 15.00% to 20.00% of the total number of pregnant women deliver through Caesarean section yearly and within these years drop to as low as 5.00%.

## 2 Methods and materials

Several studies have been conducted on the issues affecting normal delivery, still birth incidences and epidemiology of Caesarean section child delivery among women in Nigeria [1], [2], [3], [4], [5], [6], [7], [8], [9], [10], [11], [12], [13], [14], [15], [16], [17], [18], [19]. Similar data articles on medicine that applied statistical tools could be helpful, readers are refer to [20], [21], [22], [23], [24], [25], [26], [27], [28], [29].

Correlation and time series tools are used to explore the data of child delivery in Akure, Nigeria. Pearson correlation coefficients were calculated for the each pairs of total delivery, normal delivery, still birth and Caesarean section. Furthermore, autoregressive integrated moving average (ARIMA) was used in describing and modeling the pattern of child delivery. The correlation was done using the Microsoft Excel while the time series analysis was done with the aid of the R software.

### 2.1 Correlational study

The correlation coefficient shows the degree of linear relationship that exists between two variables; this was presented in Table 2. There is a very high correlation between total and normal delivery (0.98098), followed by normal delivery and still birth (0.64250), while the least is between Caesarean section and still birth (0.24032).

### 2.2 Autoregressive integrated moving average (ARIMA)

ARIMA is a time series statistical tool used in describing and modeling the pattern of a given seasonal and non-seasonal time series data. Table 3, Table 4, Table 5, Table 6 present the appropriate ARIMA models for each of the indicators under consideration. It was observed that ARIMA (0, 1, 1) is best for describing and forecasting the future counts for three of the indicators: total delivery, normal delivery and still birth, while ARIMA (3, 0, 0) is most appropriate for the number of delivery through Caesarean section.
