# Supplementary material for: Quantitative evaluation of pregnant women delivery status’ records in Akure, Nigeria
Source: Data Brief. 2017 Nov 14;16:127–34. doi: 10.1016/j.dib.2017.11.041 (PMC5699871; doi:10.1016/j.dib.2017.11.041)
Supplement: Supplementary file 1 — Supplementary material [file mmc1.pdf]

COVENANT UNIVERSITY
COLLEGE OF SCIENCE AND TECHNOLOGY
DEPARTMENT OF MATHEMATICS

CANAANLAND, KM 10, IDIROKO ROAD
P.M.B 1023, OTA, OGUN STATE, NIGERIA
www.covenantuniversity.edu.ng, mat.covenantuniversity.edu.ng

EXTERNAL MEMO

To: Editor, Data in Brief
From: Corresponding Author
Date: 15th October, 2017
Subject: **Conflict of Interest**

I thereby declare the absence of any conflict of interest among the authors.

The authors have read the final draft and unanimously agreed that the paper be sent for review.

The source of funding and data have been acknowledged.

Hilary I. Okagbue
Department of Mathematics, Covenant University, Ota.
Google Scholar: Hilary Izuchukwu Okagbue
Research Gate: Hilary Okagbue
Live DNA: 234.14453
Scopus ID: 56438006100
ORCID: 0000-0002-3779-9763
